# Severe Respiratory Distress From Subcutaneous Emphysema After Tracheocutaneous Fistula Repair: A Case Report

**DOI:** 10.7759/cureus.76328

**Published:** 2024-12-24

**Authors:** David P Fitzgerald, Megan L Gray, Arundathi Reddy, Destiny F Chau

**Affiliations:** 1 Pediatric Anesthesiology, University of Arkansas for Medical Sciences, Little Rock, USA; 2 Biology, Hendrix College, Conway, USA

**Keywords:** airway obstruction, ent complication, pacu, respiratory distress, tcf closure

## Abstract

Subcutaneous emphysema results from air or gas being forced into the fascial spaces of subcutaneous tissue. Once the air or gas has entered the fascial spaces, it travels along connective tissue causing a mass effect and swelling. This rare complication usually presents with mild severity during the immediate postoperative period following surgical procedures of the head or neck regions and self-resolves with conservative treatment. However, a range of presentations are possible to the point of being life-threatening, as in the present case in which the mass effect on the airway was severe. This report focuses on a life-threatening case of subcutaneous emphysema in a three-year-old male patient who was recovering in the postanesthesia care unit following tracheocutaneous fistula closure. Prompt recognition of the root cause, urgent placement of a new tracheostomy tube for restoration of the airway, and administration of sedation to minimize the amount of air forced into the fascial spaces were crucial for a safe patient outcome. This discussion highlights the importance of awareness of this complication, timely rescue management, and strategies for intraoperative anesthetic management to prevent and mitigate triggering factors.

## Introduction

Subcutaneous emphysema leads to swelling and tissue displacement, manifesting as crepitus on palpation. It has been reported to occur in relation to surgical procedures of the head and neck, including dental procedures, facial bone surgeries, tracheostomies, tracheocutaneous fistula (TCF) closure, thoracic injuries, and esophageal or hypopharyngeal perforations [1,2]. The incidence of subcutaneous emphysema ranges from 0.43 to 2.34% [3]. Mechanisms of air entry into fascial spaces include direct openings such as injuries to the respiratory or alimentary tract or dissection from one fascial plane to another. Additional activities such as coughing, crying, nose blowing, positive pressure ventilation, vomiting, inhaling, or using a straw can further force air into fascial spaces potentially worsening this condition [2,4].

TCFs occur after tracheostomy decannulation. While 50% of TCFs close spontaneously in children, the remaining 50% persist for months necessitating surgical closure. Migration of epithelial cells into the tracheostomy stoma site prevents tract closure [5,6]. Persistent TCFs can hinder bathing and swimming, increase risk for aspiration, infection, and skin excoriation, and have associated negative aesthetics and social impact. Risk factors for TCF persistence include younger age at placement and longer duration of tracheostomy cannulation [5,6]. Surgical closure of TCFs has a low risk of acute serious complications [5,6].

## Case presentation

We report a rare case of subcutaneous emphysema with airway obstruction. A three-year-old male patient presented for microlaryngoscopy, bronchoscopy, and TCF closure. The patient was born prematurely at 24 weeks, was ventilator-dependent, and subsequently had a tracheostomy placed at seven months of age. He was weaned off the ventilator at two years old. His past medical history was significant for chronic lung disease of prematurity, developmental delay, gastroesophageal reflux disease, and gastrostomy tube feeding dependency. The patient had previously undergone six microscopy and bronchoscopy (MLB) procedures as well as multiple other procedures without perioperative complications. He had been decannulated six months prior to the surgery of the incident, and the TCF still remained open.

**Figure 1:**
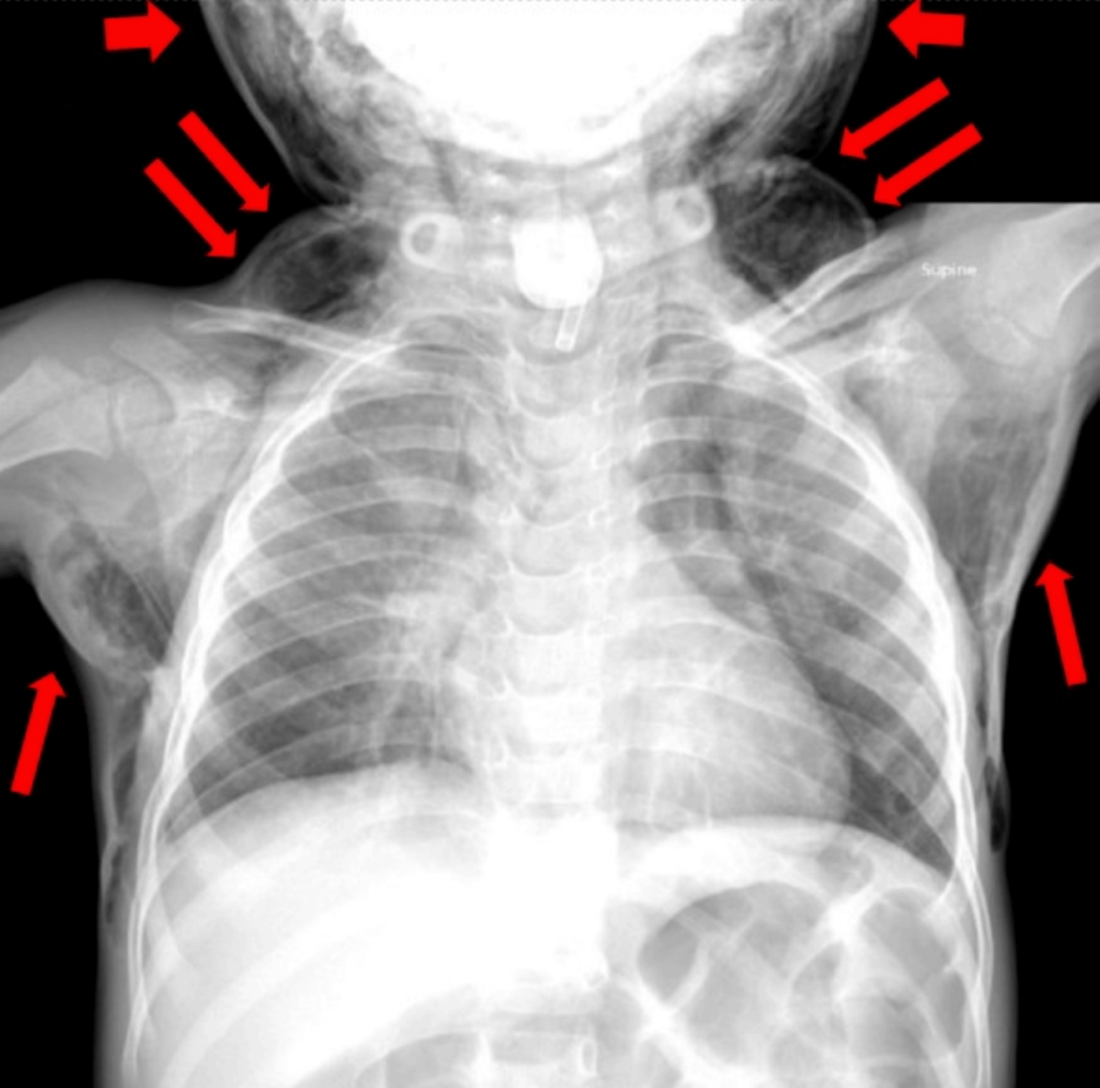
Frontal supine radiograph of the chest revealing severe subcutaneous emphysema of the soft tissues of the face, neck, and chest wall (red arrows). The mass effect of the subcutaneous emphysema resulted in severe airway obstruction.

On preoperative evaluation, the patient had normal vital signs, met nil per os criteria, and was medically optimized. After obtaining informed consent, preoperative anxiolysis was administered with midazolam via the gastrostomy tube, and the patient was transported to the operating room. General anesthesia was induced via mask induction with sevoflurane, followed by insertion of a peripheral intravenous catheter and initiation of propofol infusion for maintenance of anesthesia (titrated between 200 and 300 mcg/kg/min). Spontaneous ventilation was maintained via a natural airway. Additionally, IV dexamethasone (0.5 mg/kg bolus) and dexmedetomidine (titrated to a total of 0.5 mcg/kg) were administered. Microlaryngoscopy and bronchoscopy revealed no fistula tract skin entering the trachea, and the trachea could accept a 4.0 cuffed endotracheal tube. The surgical technique used for closing the TCF was partial closure with healing by secondary intent. The wound was partially closed laterally to medially. The surgeon inserted a 2.5 pediatric cuffless tracheostomy tube to keep the tract open until the patient recovered from anesthesia, with orders for subsequent removal in the recovery unit. The procedure was uneventful and the patient was transferred to the post-anesthesia care unit (PACU) and was breathing spontaneously with blow-by oxygen with a pulse oximetry (SpO2) reading of 96%. The small tracheostomy was removed once the patient was comfortable and hemodynamically stable. Shortly thereafter, the patient started to cry with ensuing hypoxia and respiratory distress. The SpO2 had quickly decreased to 85%. It was noted that the tongue was enlarged, with rapidly increasing swelling of the eyes, face, neck, and chest. Extensive crepitus was palpated. Emergently, the otorhinolaryngologist placed an angiocath into the repaired TCF site followed by a 2.5 cuffless tracheostomy tube. Concurrently, dexmedetomidine boluses along with infusion were titrated for sedation. These maneuvers resulted in improved oxygenation and ventilation. A chest radiograph was then obtained showing subcutaneous emphysema of the soft tissues of the face, neck, and chest wall, with no obvious evidence of pneumothorax in this initial chest radiograph (Figure 1).

The patient was admitted to the pediatric intensive care unit (PICU) for overnight monitoring. By the following day, as the subcutaneous emphysema began to resolve, the tracheostomy tube was removed uneventfully. The patient was transferred to the inpatient unit for further observation. He progressed well and was discharged from the hospital on the fourth postoperative day.

## Discussion

Subcutaneous emphysema is a rare but known complication of TCF repair that often presents in the immediate postoperative period. Although the presence of subcutaneous air in itself is not damaging, it can be life-threatening if it causes a mass effect such as airway obstruction, pneumomediastinum, pneumocardium, and pneumothorax leading to cardiac arrest. Therefore, it is vital that patients at risk are closely monitored for this specific complication.

Signs and symptoms of subcutaneous emphysema include difficulty breathing, dyspnea, dysphagia, swelling, sore throat, cough, respiratory difficulties, and crepitus of the skin [7]. For patients with subcutaneous emphysema, crepitus of the skin can be identified as a crackling sound or sensation that may be auscultated or palpated. There are several mechanisms by which air can enter the subcutaneous tissues. A common mechanism includes air being forced into open surgical repair sites, such as perforations from tracheostomy tubes, direct openings of skin lacerations, or mucosal injuries. Pulmonary alveolar rupture from trauma, or the pressure gradient, allows the pressurized alveolar gas to burst through the alveolar membrane and allows the gas to track along fascial planes into the subcutaneous tissues [8]. In dental procedures, the use of pressurized air to dry the exposed dental surfaces has been reported to lead to severe subcutaneous emphysema [4]. Since the fascial planes of the neck connect with the mediastinum and retroperitoneum, the displaced air can spread along these body sectors and cause significant cardiopulmonary compromise [8].

A patient’s actions such as coughing, crying, vomiting, straining against a closed glottis (Valsalva maneuver), blowing the nose, or blowing against resistance pressurize the air to enter into surgical openings [9,10]. Additionally, healthcare providers can cause air to enter a patient’s fascial spaces if excessive positive pressure ventilation or pressurized air is used following a surgical procedure of the neck or head. In a narrative literature review of ear nose and throat procedures, Cerritelli et al. found that subcutaneous emphysema, although rare, has been reported to occur after nearly all ENT procedures: orotracheal intubations, nasal, laryngotracheal, thyroid, and ear and adenotonsillectomy surgeries, with tracheal surgeries being the most common at 1.4% incidence after tracheostomy [11,12].

The optimal surgical technique for TCF closure to prevent air leaks is still debated. Wong et al. reported a subcutaneous emphysema complication rate of 2 in 67 (3%) children after simple excision of the tract with primary closure in layers with a subcutaneous drain left for air to escape [5]. Another technique is partial closure and healing with secondary intent, allowing for air exit. The evidence does not clearly favor one technique over the other [5]. Patient factors may also play a role such as asthma, reactive airway disease, and chronic lung disease, which may predispose a patient to paroxysms of coughing. A history of nausea and vomiting or emergence delirium can also exacerbate the triggering actions of emesis, Valsalva, crying, and coughing.

The differential diagnosis of rapid swelling of acute onset includes bleeding, necrotizing infections with production of air, and edema from anaphylaxis or other related immune reactions. Diagnosis of subcutaneous emphysema is crepitus on palpation and X-ray findings. Mild subcutaneous emphysema typically has a benign clinical course, with conservative treatment being sufficient for its resolution. Prevention of further air accumulation is essential. Air entrapped within soft tissues gets reabsorbed with complete resolution usually occurring within two to five days. Administration of 100% oxygen increases the rate of nitrogen resorption. Prevention of cough, nausea and vomiting prophylaxis, adequate pain control, and emergence delirium prevention are treatments with a favorable benefit to risk profile. Patients after TCF closure are routinely admitted overnight for observation [13].

In our case, the patient’s agitation and crying likely forced air into the partially closed tracheostomy repair site, leading to subcutaneous emphysema. Once air entered the wound, it traveled into the fascial spaces of the subcutaneous tissue and resulted in swelling of the eyes, face, neck, chest wall, and tongue, leading to airway obstruction, respiratory distress, cyanosis, and further agitation. Crepitus of the skin was evident on palpation. Recognition and restoration of airway patency via the re-insertion of a small tracheostomy, and administration of supplemental oxygen and sedation stabilized the patient.

Anesthesiologists play a central role in the prevention of subcutaneous emphysema by reducing triggers that contribute to subcutaneous emphysema, for example, administering antiemetics, maintaining adequate pain control, reducing emergence delirium, minimizing the risk of laryngospasm, and suctioning secretions before emergence of anesthesia to prevent coughing. Early recognition of subcutaneous emphysema for risk stratification and proper management is paramount. Mild cases of subcutaneous emphysema can be managed conservatively with analgesics, supplemental oxygen, and supportive care [14]. Severe subcutaneous emphysema should be treated more aggressively as it can be life-threatening. Although the occurrence of subcutaneous emphysema is rare, it can become catastrophic very quickly, therefore, it is important for anesthesiologists to be aware of this complication and be prepared to rescue. During rescue, excessive levels of positive pressure ventilation should be avoided as it can exacerbate this condition. Subcutaneous emphysema can lead to airway obstruction and distortion of the airway, making endotracheal intubation challenging [15]. The study by Wong et al. reported that two patients, after multilayer closure and drain left in place, developed catastrophic subcutaneous emphysema; both patients were reintubated urgently and kept intubated for further management [5]. Following partial closure of the TCF, definitive airway placement via tracheostomy minimized the need for positive pressure ventilation via mask or endotracheal tube placement.

## Conclusions

This case highlights the critical importance of intraoperative anesthetic planning to minimize factors that could contribute to subcutaneous emphysema. Key strategies include preventing coughing, providing prophylaxis for nausea and vomiting, ensuring adequate pain control, and addressing emergence delirium to reduce triggers that may exacerbate subcutaneous emphysema. Multimodal postoperative pain management and the use of medications such as dexmedetomidine boluses intraoperatively can help reduce agitation during recovery. As this complication occurs mainly during the postoperative period, awareness and vigilance in the PACU for prompt airway interventions are essential for good patient outcomes.
